# Contribution of Amygdala Histone Acetylation in Early Life Stress-Induced Visceral Hypersensitivity and Emotional Comorbidity

**DOI:** 10.3389/fnins.2022.843396

**Published:** 2022-05-06

**Authors:** Le Guan, Xi Shi, Ying Tang, Yan Yan, Liang Chen, Yu Chen, Guangcheng Gao, Chun Lin, Aiqin Chen

**Affiliations:** ^1^Fujian Provincial Key Laboratory of Brain Aging and Neurodegenerative Diseases, School of Basic Medical Sciences, Pain Research Institute, Fujian Medical University, Fuzhou, China; ^2^Department of Medical Oncology, The First Affiliated Hospital of Fujian Medical University, Fuzhou, China; ^3^Department of Pediatrics, The First Affiliated Hospital of Fujian Medical University, Fuzhou, China

**Keywords:** amygdala, histone acetylation, histone deacetylase, protein kinase Mζ, early life stress (ELS)

## Abstract

Patients with irritable bowel syndrome (IBS) experience not only enhanced visceral pain but also emotional comorbidities, such as anxiety and depression. Early life stress (ELS) is a high-risk for the development of IBS. Literatures have reported an important epigenetic modulation in sustaining extrinsic phenotypes. The amygdala is closely related to the regulation of visceral functions and emotional experiences. In this study, we hypothesized that ELS-induced reprogramming inappropriate adaptation of histone acetylation modification in the amygdala may result in visceral hypersensitivity and anxiety-like behaviors in ELS rats. To test this hypothesis, the model of ELS rats was established by neonatal colorectal dilatation (CRD). Visceral hypersensitivity was assessed based on the electromyography response of the abdominal external oblique muscle to CRD. Emotional comorbidities were examined using the elevated plus maze test, open field test, and sucrose preference test. Trichostatin A (TSA) and C646 were microinjected into the central amygdala (CeA) individually to investigate the effects of different levels of histone acetylation modification on visceral hypersensitivity and emotion. We found neonatal CRD resulted in visceral hypersensitivity and anxiety-like behaviors after adulthood. Inhibiting histone deacetylases (HDACs) in the CeA by TSA enhanced visceral sensitivity but did not affect anxiety-like behaviors, whereas inhibiting HAT by C646 attenuated visceral hypersensitivity in ELS rats. Interestingly, CeA treatment with TSA induced visceral sensitivity and anxiety-like behaviors in the control rats. Western blot showed that the expressions of acetylated 9 residue of Histone 3 (H3K9) and protein kinase C zeta type (PKMζ) were higher in the ELS rats compared to those of the controls. The administration of the PKMζ inhibitor ZIP into the CeA attenuated visceral hypersensitivity of ELS rats. Furthermore, the expression of amygdala PKMζ was enhanced by TSA treatment in control rats. Finally, western blot and immunofluorescence results indicated the decrease of HDAC1 and HDAC2 expressions, but not HDAC3 expression, contributed to the enhancement of histone acetylation in ELS rats. Our results support our hypothesis that amygdala-enhanced histone acetylation induced by stress in early life results in visceral hypersensitivity and anxiety-like behaviors in ELS rats, and reversing the abnormal epigenetic mechanisms may be crucial to relieve chronic symptoms in ELS rats.

## Introduction

Patients with irritable bowel syndrome (IBS) experience not only enhanced visceral pain and abnormal bowel habits but also emotional comorbidities, such as anxiety and depression (Pimentel and Lembo, 2020). Visceral hypersensitivity and negative emotions of patients with IBS persist in a long-term and aggravate each other. Thus, the treatment for IBS in clinics is considered challenging (Vierck et al., 2014). However, whether visceral hypersensitivity of IBS shares the same molecular pathways with negative emotions or not remains unclear.

Patients with IBS are two to four times more likely to report a history of early life stress (ELS), such as abuse, poverty, or trauma, in the early stages than healthy controls (Bradford et al., 2012). ELS is a high-risk for the development of IBS. Literatures have reported an important epigenetic modulation in sustaining extrinsic phenotypes (Mitrousis et al., 2015; Louwies and Greenwood-Van Meerveld, 2020; Marcal et al., 2021). For example, histone acetylation modification can regulate gene expression and result in long-term abnormal behaviors (Graff and Tsai, 2013). Epigenetic modifications in the CeA serve as memories of adverse events that occurred during early life (Louwies and Greenwood-Van Meerveld, 2020). The level of histone acetylation is dependent on the activities of histone deacetylases (HDACs) and histone acetyltransferases (HATs) (Bahari-Javan et al., 2014). Studies have shown that HDAC inhibitors (HDACIs) could alleviate neuropathic pain (Khangura et al., 2019). HDACIs showed analgesic effects in animal models of inflammatory persistent pain (Mao et al., 2019). The upregulation of HDACs was also involved in chronic pain caused by bone cancer through the pathological activation of microglia and astrocytes in the spinal dorsal horn (Hu et al., 2017). On the other hand, C646, an inhibitor of HAT p300, attenuated mechanical allodynia and thermal hyperalgesia in the spinal cord (Mao et al., 2019). Furthermore, administration of the HAT inhibitor garcinol into central amygdala attenuated ELS-induced visceral hypersensitivity in adult female rats (Louwies and Greenwood-Van Meerveld, 2020). These studies have indicated that histone acetylation modification is correlated with chronic pain.

As an important part of the cortex–limbic network, the amygdala is closely related to the regulation of visceral functions, emotional experiences, and fear memories (Gauriau and Bernard, 2004; Chaloner and Greenwood-Van Meerveld, 2013; Lucassen et al., 2014; Cheng et al., 2019). The injection of HDAC inhibitor trichostatin A (TSA) into the lateral amygdala of rats enhanced H3 acetylation and auditory fear memory consolidation, whereas HAT inhibitor destroyed the consolidation and reconsolidation of Pavlovian fear conditioning ability (Maddox et al., 2013a). Microinjection of HDACIs into the central amygdala attenuated anxiety-like behaviors and hypersensitivity reactions caused by increased corticosteroid exposure (Tran et al., 2015). Above all, the amygdala is certainly involved in the regulation of pain and emotion, but little is known about the roles of histone acetylation modification in regulating visceral hypersensitivity and negative emotions of ELS rats. We previously found that the expression of protein kinase C zeta type (PKMζ) in the hippocampus of IBS rats increased (Chen et al., 2015; Tang et al., 2016). Zeta inhibitory peptide (ZIP), an inhibitor of PKMζ, could attenuate chronic visceral hypersensitivity in IBS rats (Chen et al., 2015; Tang et al., 2016). Studies have shown that amygdala PKMζ was involved in the regulation of stress and fear memory (McDonald and Mott, 2017; Zhou et al., 2018). However, elucidating the roles of amygdala PKMζ and the association between histone acetylation modification and PKMζ expression in ELS rats is required.

The primary aim of this study was to determine the roles of histone acetylation modification and HDAC1–3 in regulating visceral hypersensitivity and negative emotions of ELS rats. The secondary aim was to examine PKMζ expression and roles in the amygdala of ELS rats and to determine whether it is regulated by histone acetylation modification. This study could reveal novel underlying mechanism of IBS and provide new molecule targets for the treatment of IBS.

## Materials and Methods

### Animal

Male Sprague–Dawley rats were purchased from the Laboratory Animal Center of Fujian Medical University. The approval number is SCXK (Fujian) 2012-0001. ELS rats were established by colorectal dilation (CRD) stimulation of 60 mmHg pressure for 1 min using a 2.5 mm × 20 mm human vascular reconstruction balloon (Chen et al., 2014; Fan et al., 2021). The stimulation was performed once a day from the 8th to the 14th day after birth. The pups were with dams until 21 days old. Control rats were fed normally under the same conditions (a 12-h light/dark cycle with *ad libitum* access to food and water) without neonatal colorectal dilation. The animal procedures were approved by the Committee for Care and Use of Laboratory Animals of Fujian Medical University. Behavioral and molecular biological experiments were carried out at postnatal 8 weeks. Experiments were performed by researchers blinded to the treatment of animals.

### Assessment of Visceral Hypersensitivity

Male rats (8 weeks old) were anesthetized with light isoflurane. Abdominal electromyography (EMG) response to CRD stimulation was measured to assess visceral hypersensitivity as previously described (Chen et al., 2014; Fan et al., 2021). CRD induces abdominal contractions, and this visceromotor response is used to assess visceral pain. EMG responses were measured with a system of RM6240BD (Chengdu, China). Two silver electrodes were inserted into the abdominal muscles to record EMG, which was induced by graded CRD of 40 or 60 mmHg for 10 s at intervals of 4 min. ΔEMG amplitude was determined as the following formula.

ΔEMG amplitude = (EMG amplitude of CRD-EMG amplitude of baseline) × 100%

/EMG amplitude of baseline.

### Assessment of Anxiety-Like and Depression-Like Behaviors

The elevated plus-maze test (EPMT) was used to score anxiety-like behaviors in rats before and 24 h after the final drug treatment. A video camera was located above the EPM to record the behavior of each rat for 5 min (Yang et al., 2019). The video was then analyzed by an investigator who was blind to the treatment. Rats were placed in the experimental room for 30 min to adapt to the environment and were then placed in the center of the EPM facing an open arm. The percentage of time spent in the open arms and the number of open arm entries were used to quantify anxiety-like behaviors. Reduced open arm exploration and entries indicates higher level of anxiety.

The open field test (OFT) was used to assess locomotor and anxiety-like behaviors of rats (Liu et al., 2021). The open field was a large square arena with a side length of 100 cm and 40-cm-high walls in a dimly lit room. The activity of rats in the OFT was videotaped during a 5-min session and later tracked using an IR color dome camera (MODEL: TA-758RP, Shanghai Yishu Information Technology Co., Ltd., China) (Yang et al., 2019; Farazi et al., 2021). We measured the indicators including the total distance traveled, the distance and time spent in the center area, and the number of standings. Less center area exploration and standing activity indicates anxious emotion.

Sucrose preference test (SPT) was used to examine anhedonia in rats, which is the core manifestation of depression. The rats were single-housed in the cages and provided two bottles of water both containing 150 ml of 1% sucrose solution for the first 24 h. For the next 24 h, two bottles of water were supplied containing pure water and 1% sucrose solution, respectively. After the adaptive period, the rats were deprived of food and water for the next 24 h. Finally, each rat was free to access a bottle of 1% sucrose solution and a bottle of pure water at the same time, and sucrose preference was examined for 1 h (Zhang et al., 2021). The two bottles were repositioned midway during the test time to avoid position preference in drinking behaviors. The consumption of water and sucrose was measured by the weight difference of the water bottles before and after the test. Sucrose preference was calculated as sucrose intake/(sucrose intake + water intake) *100%.

### Surgical Procedures

After isoflurane (Shandong Keyuan Pharmaceutical Co., Ltd., China) anesthesia, rats were fixed in a stereotaxic instrument (Narishige, Japan) for the stereotaxic implantation of a catheter. A small cut was made to expose the skull using aseptic techniques, and 1 mm holes were drilled −3.3 mm posterior and ± 4.8 mm lateral to the bregma. Bilateral catheters were lowered −7.5 mm from the dura.

#### Stereotaxic Implantation of Cannula for CeA Infusions

Implantation of cannula was carried out as previously described (Tran et al., 2015). A custom indwelling cannula, injector, and cannula cap were provided by Shenzhen RWD Life Science (RWD, Shenzhen, China). Each cannula (inside diameter: 0.34 mm; outer diameter: 0.48 mm), which extended 7.5 mm below the threaded pedestal, was placed flush to the skull at the same coordinates. A stainless steel screw was mounted triangularly to the two sides of the cannula and held to the skull with adhesive. The rats recovered without disturbance for 1 week. TSA [200 μmol/L (Wei et al., 2016); Meilunbio, Dalian, China], C646 [2 mmol/L (Maddox et al., 2013b); MedChem Express, China], PKMζ inhibitor ZIP [0.5 μmol/L (Chen et al., 2015); ab120993, Abcam] or vehicle (VEH) (0.1% dimethyl sulfoxide) was administered once. Isoflurane 2% was used to anesthetize rats through the anesthetic mask. Then the matched injector was placed into the cannula. We injected a total volume of 1 μl of TSA, C646, ZIP or VEH into each cannula at a rate of 0.05–0.1 μl min^–1^ for a total of 10–20 min using a microinjector (Shanghai Gaoge Industry and Trade Co., Ltd., China). The injector was kept in place for an additional 10 min to ensure complete diffusion of the solvent.

#### Verification of Cannula Placement

After the behavioral experiments, we microinjected bromophenol blue solution (5%) into the CeA and removed the brain for coronal section to examine the casing trajectory. The data with inaccurate location were excluded from the experimental statistics.

### Nuclear Protein Extraction

Nuclear proteins were extracted from the tissue samples using the Nuclear and Cytoplasmic Protein Extraction Kit (P0027, Beyotime Biotechnology) according to the manufacturer’s protocol. Protein quantification of the extraction product was performed using the Enhance BCA Protein Assay Kit system (P0012S, Beyotime Biotechnology). Following quantification, the samples were aliquoted and stored at −80°C for subsequent analysis.

### Western Blot Assay

The proteins from the amygdala tissue samples were separated using 10–12% gradient polyacrylamide gel (P0012A, Beyotime Biotechnology), transferred to a polyvinylidene difluoride membrane electrophoretically, and probed with Histone H3 (acetylK9) (ab10812, Abcam, 1:500), HDAC1 (ab19845, Abcam, 1:1,000), HDAC2 (ab32117, Abcam, 1:2,000), HDAC3 (ab32369, Abcam, 1:7,000), and PKM zeta (ab59364, Abcam, 1:500), respectively. β-actin (AC004, ABclonal, 1:7,000), Histone H3 (ab1791, Abcam, 1:500), and GAPDH (MB001, Bioworld, 1:5,000) were used as the controls. Horseradish-peroxidase-conjugated secondary antibody (1:10,000) was used to incubate the membrane for 8 h. Protein bands were detected using an enhanced chemiluminescence kit (WBKLS0500, Immobilon, Millipore). Protein expression indicated by the intensity of protein bands was determined using Image J software.

### Immunofluorescence Histochemical Staining

Animals were intracardially perfused with PBS and 4% paraformaldehyde. After fixation, dissected brains were cryoprotected in 20% sucrose overnight at 4°C and then in 30% sucrose at 4°C. Coronal sections of 20 μm were obtained using a cryostat and further processed for immunohistochemistry. Briefly, slices were incubated in blocking fluid (Beyotime) to permeabilize and block unspecific staining. Primary antibodies were incubated for 48 h at 22–24°C and secondary antibodies overnight at 4°C. Both primary and secondary antibodies were diluted in blocking fluid (Beyotime). Rabbit anti-HDAC1 (ab19845, Abcam, 1:1,000), anti-HDAC2 (ab32117, Abcam, 1:250), and anti-HDAC3 (ab32369, Abcam, 1:100) were used as primary antibodies. All secondary antibodies Alexa 488 (Invitrogen) was diluted 1:1,000. Immunofluorescence labeling of a specific protein was used to determine its localization with the cells. Immunofluorescence images were captured using laser scanning confocal microscope (FV3000, Olympus, Japan). Left or right CeA was imaged. Integrated optical density (IOD) of the specific protein was analyzed using the Image-Pro Plus 6.0 software (Media Cybernetics, United States). The relative concentration of the labeled protein was quantified by specific thresholding of the fluorescent region of interest and by measuring the IOD of the fluorescent signal.

### Statistical Analysis

SPSS 17.0 software was used for data analysis. Data are presented as mean ± SEM. The data for EMG and behaviors were analyzed with one-way ANOVA and independent samples *t*-test. Western blotting and Immunofluorescent data were analyzed with one-way ANOVA and independent samples *t*-test. The administration of TSA, ZIP or C646 was compared using a paired-samples *t*-test. Statistical analysis was performed using GraphPad Prism 8.0. Immunofluorescent graphs were quantified by integrated optical density (IOD) with the Image-Pro Plus 6.0 software. *P* < 0.05 was considered statistically significant. If the distribution of data was non-normal or the variance was uneven, the non-parametric test (Mann–Whitney *U* test or Wilcoxon signed-rank test) was used.

## Results

### Neonatal Colorectal Distension Resulted in Visceral Hypersensitivity and Anxiety-Like Behaviors in Adult Rats

**FIGURE 1:**
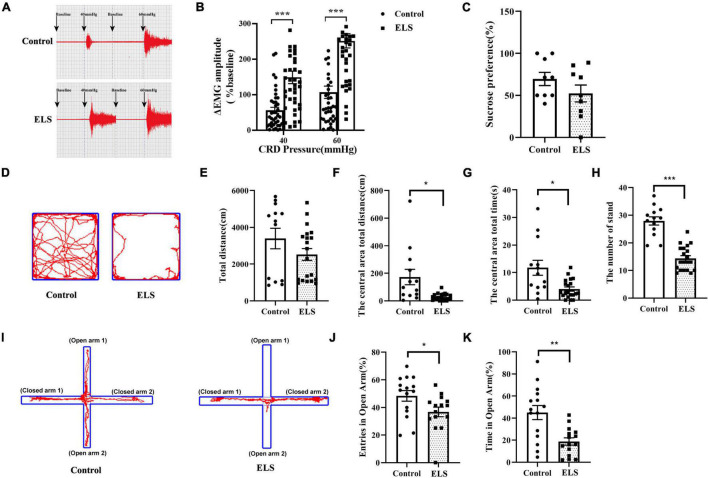
Neonatal CRD resulted in visceral hypersensitivity and anxiety-like behaviors in adult rats. **(A,B)** Successful chronic visceral pain modeling with EMG assessment. The original typical traces of EMG **(A)**. Amplitude of ΔEMG in ELS rats, compared to that of controls, shown by quantification **(B)**. *N* = 40. One-way ANOVA and Mann–Whitney *U* test were performed. **(C)** Sucrose preference test used to detect depression-like behavior in rats. *N* = 9. Independent samples *t*-test was performed.**(D–H)** Anxiety-like behaviors were tested in the OFT. Original traces of OFT **(D)**. Quantification of the total distance **(E)**, the central area total distance **(F)**, the central area total time **(G)**, and the number of standings in OFT **(H)**. *N* = 13–20. Independent samples *t*-test was performed. **(I–K)** Anxiety-like behaviors were tested in the EPMT. Original trajectory representation diagram of EPMT **(I)**. Quantification of entries **(J)** and time **(K)** in the open arms. *p < 0.05, **p < 0.01, ***p < 0.001. *N* = 15. Independent samples *t*-test was performed **(J)**.One-way ANOVA and Mann–Whitney *U* test were performed **(K)**. N represents the number of rats per group.

Electromyography (EMG) amplitudes were significantly higher at 40 and 60 mmHg colorectal distension (CRD) pressure in ELS rats than in control rats (Figures 1A,B). Anxiety-like behaviors were examined using the elevated plus maze test (EPMT) and open field test (OFT) in rats. In the OFT, the distance, time spent in the center area, and the number of standings were lower in ELS rats than in control rats (Figures 1D, F–H), while the total distance did not change significantly (Figure 1E). Similar results were found in the experiments using the EPMT. Time spent in the open arms and entries into the open arms of ELS rats were significantly less than those of control rats (Figures 1I–K). Anhedonia was also measured using the sucrose preference test. However, there was no significant reduction in the sucrose preference ratio of ELS rats relative to that of control rats (Figure 1C). These results indicated that neonatal CRD resulted in visceral hypersensitivity and anxiety-like behaviors in adult rats.

### The Effects of Central Amygdala Treatment of Trichostatin A or C646 on Visceral Hypersensitivity and Anxiety-Like Behaviors of Early Life Stress Rats

Chronic phenotypes can be sustained by epigenetic mechanisms, such as histone modifications. To clarify the contribution of histone acetylation to visceral hypersensitivity and anxiety-like behaviors of ELS rats, TSA and C646 were microinjected into the central amygdala (CeA) individually. Localization of drug placement and verification of the location accuracy of CeA were performed following bromophenol blue staining of microinjection. Those with inaccurate positioning were discarded.

**FIGURE 2:**
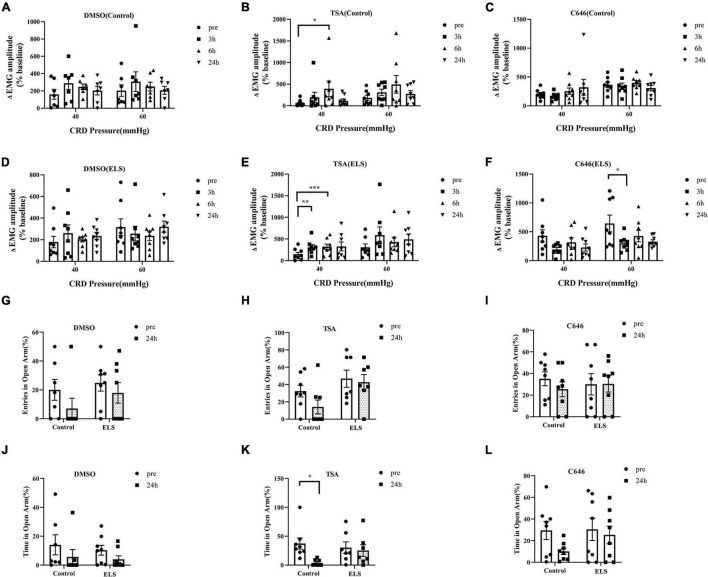
Effects of TSA and C646 on pain and anxiety-like behaviors in rats. **(A–F)** Quantification of the amplitude of ΔEMG response to CRD (40, 60 mmHg) before and after microinjection of DMSO **(A)**, TSA **(B)**, C646 **(C)** in control rats and DMSO **(D)**, TSA **(E)**, C646 **(F)** in ELS rats. **(G–I)** Quantification of entries in the open arms before and after the microinjections of DMSO **(G)**, TSA **(H)**, and C646 **(I)** in rats. **(J–L)** Quantification of time in the open arms before and after the microinjections of DMSO **(J)**, TSA **(K)**, and C646 **(L)** in rats. *p < 0.05, **p < 0.01, ***p < 0.001. *N* = 7–8. A paired-samples *t*-test was performed **(A,G–J,L)**. A paired-samples *t*-test and Wilcoxon signed-rank test were performed **(B–F,K)**. N represents the number of rats per group.

Overall, significant effects of HDACI (TSA) infusion on EMG and open arms time of the EPMT in ELS rats were observed. DMSO had no effect on EMG and EPMT (Figures 2A,D,G,J). Treatment with TSA significantly enhanced visceral hypersensitivity at 3 h and 6 h in ELS rats (Figures 2B,E), whereas it was only at 6 h in control rats. The entries in the open arms of EPMT were not significantly changed by TSA treatment in control and IBS rats (Figure 2H). Interestingly, treatment with TSA resulted in a significant decrease in the time spent exploring the open arms of the EPMT (Figure 2K) only in control rats. Our results suggested that CeA treatment with TSA induced visceral hypersensitivity and anxiety-like behaviors in control rats and enhanced visceral hypersensitivity in ELS rats.

C646, a HAT inhibitor, was expected to have an opposite effect of TSA by increasing histone acetylation. Expectedly, the microinjection of C646 in the CeA attenuated visceral hypersensitivity of ELS rats when compared with control rats (Figures 2C,F). However, treatment of C646 did not affect anxiety-like behaviors of ELS rats in the experiment of the EPMT (Figures 2I,L).

### The Involvement of Acetylated Lysine 9 Residue of Histone 3 (acH3K9) and Protein Kinase C Zeta Type in Early Life Stress Rats

**FIGURE 3:**
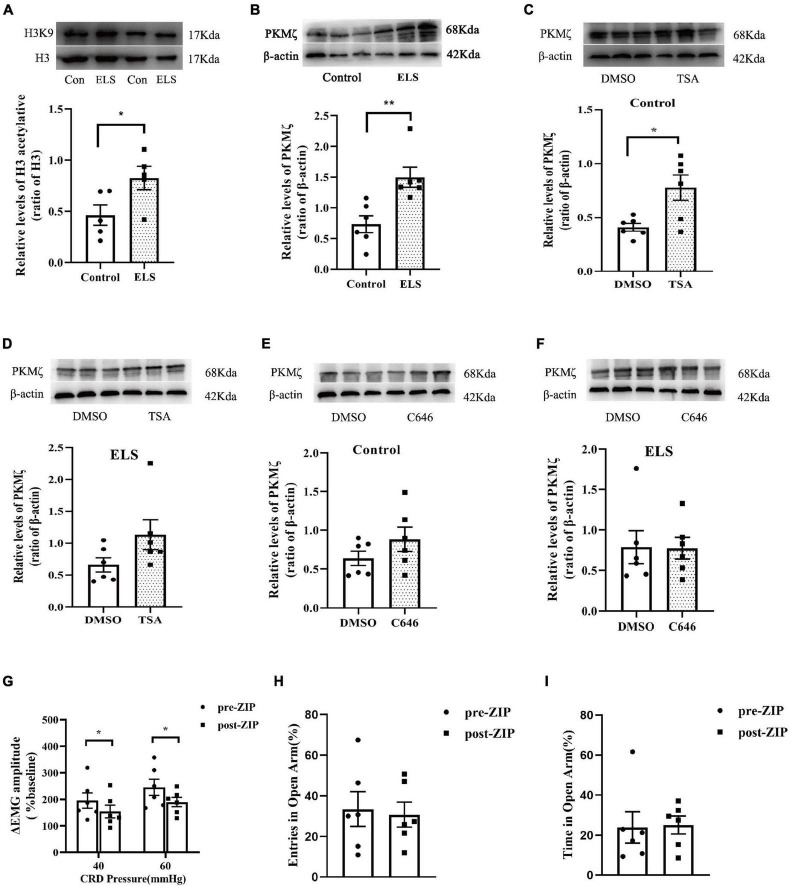
The expression of acH3K9 and PKMζ in the amygdala of rats. And effects of ZIP on visceral hypersensitivity and anxiety-like behaviors in rats. Western blot showed that the expression of AcH3 **(A)** and PKMζ **(B)** increased in ELS rats compared to that in the controls. *N* = 5. **(C,D)** TSA treatment of CeA enhanced PKMζ expression in the control rats **(C)** but not in the ELS rats **(D)**. **(E,F)** C646 treatment of CeA did not affect PKMζ expression in both the control **(E)** and ELS rats **(F)**. ZIP (a PKMζ inhibitor) treatment of CeA attenuated visceral hypersensitivity **(G)** without affecting entries **(H)** and time **(I)** in the open arms of the EPMT in ELS rats. *p < 0.05, **p < 0.01. *N* = 6. Independent samples *t*-test was performed **(A,C–F)**. One-way ANOVA and Mann–Whitney *U* test were performed **(B)**. A paired-samples *t*-test and Wilcoxon signed-rank test were performed **(G–I)**. N represents the number of rats per group.

Since our results indicated the contribution of histone acetylation to visceral sensitivity and anxiety-like behaviors of ELS rats, acH3K9 in the amygdala was assessed using western blot. acH3K9 expression was higher in ELS rats than in control rats (Figure 3A).

Our previous study showed that hippocampus PKMζ was involved in visceral sensitivity of ELS rats. In this study, we examined the expression of amygdala PKMζ using western blot, which was higher in ELS rats than in control rats (Figure 3B). The administration of PKMζ inhibitor ZIP into the CeA attenuated visceral hypersensitivity of ELS rats (Figure 3G). However, ZIP treatment didn’t affect anxiety-like behaviors of ELS rats in the experiment of the EPMT (Figures 3H–I).

Protein expression is possibly regulated by histone acetylation. Amygdala PKMζ expression was assessed after TSA and C646 were microinjected into the CeA individually to alter the level of histone acetylation. TSA treatment increased amygdala PKMζ expression in control rats (Figure 3C), but not in ELS rats (Figure 3D). However, C646 treatment did not affect PKMζ expression in both control and ELS rats (Figures 3E,F).

### Amygdala Histone Deacetylase 1–2 Expression Decreased in Early Life Stress Rats

**FIGURE 4:**
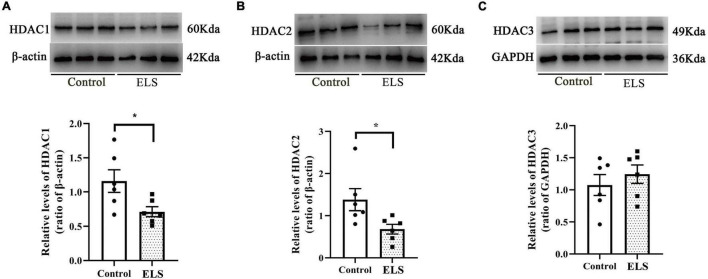
The expression of HDAC1, HDAC2 and HDAC3 in the amygdala nucleus of rats. Western blot showed that the expression of HDAC1 **(A)** and HDAC2 **(B)** in the amygdala nucleus decreased significantly while HDAC3 **(C)** expression showed no significant difference in ELS rats compared to control rats *p < 0.05. *N* = 6. Independent samples *t*-test was performed **(A–C)**. N represents the number of rats per group.

**FIGURE 5:**
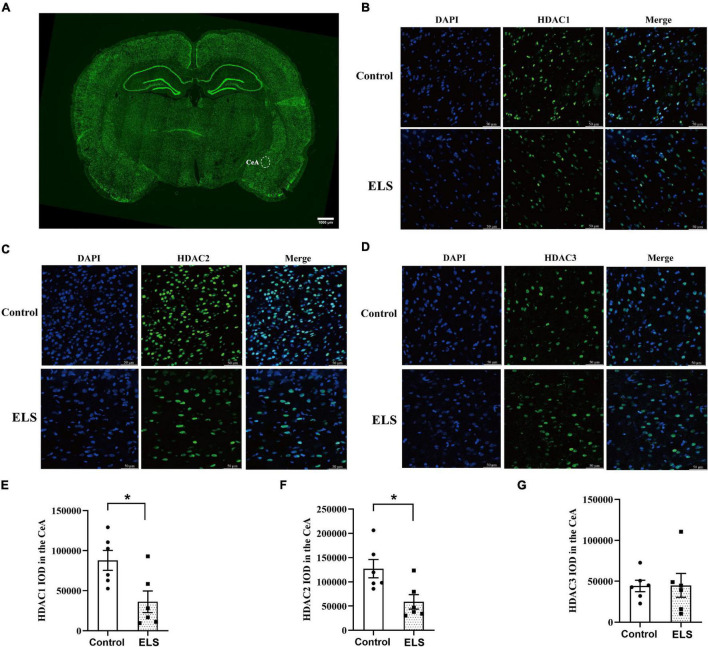
The expression and localization of HDACs in CeA. **(A)** A representative picture of brain-slice immunofluorescence (HDAC2, green) from a control rat. The scale bar is 1,000 μm. Representative confocal immunofluorescent graphs of CeA HDAC1 **(B)**, HDAC2 **(C)**, and HDAC3 **(D)** reactivity in ELS rats and control rats. IOD value of CeA HDAC1 **(E)**, HDAC2 **(F)** and HDAC3 **(G)**. The localization of CeA HDAC1, HDAC2, and HDAC3 was mainly in the nucleus (blue). The expression of CeA HDAC1 (green) and HDAC2 (green) decreased significantly in ELS rats compared to that in control rats, whereas HDAC3 (green) did not show significant difference. The scale bar is 50 μm. *p < 0.05. *N* = 6. Independent samples *t*-test was performed **(E–G)**. N represents the number of rats per group. IOD, integrated optical density.

To determine whether HDACs contributed to the elevation of histone acetylation in ELS rats, we examined the expression of nucleus HDAC1–3 in the amygdala. Amygdala nucleus HDAC1 and HDAC2 expressions decreased, whereas HDAC3 expression had no significant difference between ELS rats and control rats (Figures 4A–C). HDAC1–3 expression was further verified using immunofluorescence (Figure 5). The results are consistent with those of western blot, indicating that the decrease of amygdala nucleus HDAC1 and HDAC2 expressions, but not HDAC3 expression, contributes to the enhancement of histone acetylation in ELS rats.

## Discussion

Our study indicated the important roles of histone acetylation modification in visceral hypersensitivity and negative emotion of ELS rats. We found that neonatal CRD resulted in visceral hypersensitivity and anxiety-like behaviors after adulthood. Inhibiting HDACs in the CeA enhanced visceral sensitivity but did not affect anxiety-like behaviors, whereas inhibiting HAT attenuated visceral hyperalgesia in ELS rats. Interestingly, CeA treatment with TSA induced visceral sensitivity and anxiety-like behaviors in control rats. Western blot showed that acH3k9 and PKMζ expressions were higher in ELS rats than in control rats. The administration of the PKMζ inhibitor ZIP into the CeA attenuated visceral hypersensitivity of ELS rats. Furthermore, amygdala PKMζ expression was enhanced by TSA treatment in control rats. Finally, western blot and immunofluorescence results indicated that the decrease of HDAC1 and HDAC2 expressions, but not HDAC3 expression, contributed to the enhancement of histone acetylation in ELS rats.

Irritable bowel syndrome is characterized by repeated abdominal pain in the absence of recognizable organic pathological changes, accompanied by abnormal defecation habit (Elsenbruch, 2014; Fadgyas-Stanculete et al., 2014; Meerveld and Johnson, 2018). Animal models of IBS were often established by recapitulating early-life stress, such as mother–infant separation (Moloney et al., 2015), unpredictable electrical stimulation (Louwies and Greenwood-Van Meerveld, 2020), and neonatal CRD (Chen et al., 2014; Gil et al., 2016; Fan et al., 2021). Our previous study indicated that neonatal CRD induced visceral hypersensitivity after adulthood (Chen et al., 2014; Fan et al., 2021). In this study, we focused not only on visceral pain but also on emotional comorbidity in ELS rats. We found that neonatal CRD resulted in visceral hypersensitivity and anxiety-like behaviors after adulthood in rats. Similar results were reported in IBS rats induced by neonatal maternal separation (Moloney et al., 2015). Functional brain imaging revealed that cognitive–emotional processes had an important influence on gastrointestinal sensation (Pellissier and Bonaz, 2017). Greenwood-Van Meerveld et al. reported that patients with IBS may experience aggravation of clinical symptoms under anxiety or stress (Greenwood-Van Meerveld et al., 2016; Pimentel and Lembo, 2020). The vicious circle of visceral hypersensitivity and negative emotions is considered challenging for the treatment of IBS. IBS patients show a hyperactive amygdala in brain imaging studies (Bonaz et al., 2002). A previous study showed that decreased CeA acH3K9 expression might lead to anxiety-like behaviors and hypersensitivity reactions caused by increased corticosteroid exposure (Tran et al., 2015). We also examined acH3K9 expression in the amygdala and found neonatal CRD caused increased acH3K9 in the amygdala of adult rats. The contradicting results may be caused by different methods of establishing animal models, which involved different pathophysiological mechanisms.

Since histone acetylation is involved in ELS rats, we hypothesized that modifying the acetylation level of histones might affect visceral hypersensitivity and anxiety-like behaviors of ELS rats. Therefore, we compared visceral pain reaction and emotions before and after HDAC inhibitor TSA and HAT inhibitor C646 were microinjected into the CeA individually. TSA is an effective, non-competitive, and reversible HDAC inhibitor that can increase the level of histone acetylation modification. TSA could enhance the acetylation of histones H3K9 and H4K8 (Zhang et al., 2019) in mice. In cultured cortical neurons, TSA treatment increased the expression of H3K9 and H3K18 (Borodinova et al., 2019). In contrast, C646 is a selective and competitive HAT p300 inhibitor that has little effect on other acetyltransferases. Previous studies reported that p300 preferentially acetylates histone H3K18/K27 instead of H3K9 (Lasko et al., 2017) and deletion of p300 specifically and dramatically reduced acetylations on H3K18 and H3K27 (Jin et al., 2011). In our study, CeA treatment with TSA induced visceral hypersensitivity and anxiety-like behaviors, whereas C646 did not affect visceral pain reaction and emotional behaviors in control rats. In ELS rats, visceral hypersensitivity was enhanced by TSA and alleviated by C646. Unexpectedly, anxiety-like behaviors of ELS rats were not significantly altered by either TSA or C646. In control rats, the level of CeA acH3K9 is low and TSA treatment might increase acH3K9 level which results in visceral hypersensitivity and anxiety-like behaviors. Though ELS rats showed high level of CeA acH3K9, it seems TSA treatment could further increase acH3K9 level to aggravate visceral pain. TSA could induce visceral hypersensitivity and anxiety-like behaviors in controls and enhance visceral hypersensitivity in ELS rats, suggesting the important role of histone acetylation in ELS. The results of C646 indicate p300 might participate in the process of visceral hypersensitivity but not anxiety-like behaviors of ELS rats. Since p300 preferentially acetylates histone H3K18/K27 (Jin et al., 2011; Lasko et al., 2017), it is reasonable for us to speculate that in addition to acH3K9, there are other sites of histone acetylation involved in ELS rats. Our results are consistent with the report that intrathecal injection of HAT inhibitors could reduce the visceral hypersensitivity by colorectal dilatation (Aguirre et al., 2017). In the chronic neuropathic pain model, the decrease of HDAC subtype sirtuin-1 (SIRT1) in the CeA increased H3K9, allowing animals to be at higher risk of emotional comorbidities, such as anxiety and depression. Moreover, this emotional comorbidity could be reversed by local overexpression of SIRT1 (Zhou et al., 2020). Although the type of chronic pain is different, emotional comorbidities seem to be involved with similar histone acetylation modification. However, literatures have reported that intrathecal injection of HDAC inhibitors could attenuate visceral hypersensitivity (Cao et al., 2015, 2016; Niederberger et al., 2017). Microinjection of HDACIs into the CeA attenuated anxiety-like behaviors and hypersensitivity reactions caused by increased corticosteroid exposure (Tran et al., 2015). Inconsistent results reported in literatures have suggested that the underlying mechanisms of chronic pain and emotional comorbidities vary based on the animal model and types of pain and tissue.

Our previous studies found that the expression of PKMζ and PKMζ-dependent long-term potentiation were enhanced in the CA1 of IBS rats, and microinjection of ZIP in the hippocampus could alleviate visceral hypersensitivity (Chen et al., 2015; Tang et al., 2016), indicating that hippocampal PKMζ is involved in pain memory. However, PKMζ expression in the amygdala of IBS and the interface between histone acetylation and PKMζ are unclear. Therefore, we examined PKMζ expression and found that it was also significantly increased in the amygdala of ELS rats. Then we microinjected the PKMζ inhibitor ZIP into the CeA and found ZIP attenuated visceral hypersensitivity of ELS rats. However, ZIP treatment didn’t affect anxiety-like behaviors of ELS rats in the experiment of the EPMT. Our finding indicated the increased PKMζ in CeA might contribute to visceral hypersensitivity but not emotional comorbidity of ELS rats. PKMζ seems a key molecule involved in the pain memory induced by early life stress. PKMζ inhibition could disrupt the expression of pain memory to attenuate visceral hypersensitivity of ELS rats. In contrast, PKMζ in the CeA is not likely associated with emotional comorbidity of ELS. The functional research of the limbic brain showed that PKMζ inhibition in the basolateral amygdala, but not in the hippocampus, can disrupt fear memory (Kwapis et al., 2009). The role of PKMζ varies by brain areas and different emotional disorders. The current results might point toward two potentially different molecular mechanisms involved in the downstream of increased histone acetylation modification in CeA that modulate visceral hypersensitivity and emotional comorbidity of ELS rats. Histone acetylation and PKMζ in CeA are both involved in the visceral hypersensitivity of ELS rats. We examined if PKMζ is the molecular target of histone acetylation modification though enhanced histone acetylation in ELS rats is likely to regulate many genes. We found CeA treatment with TSA enhanced PKMζ expression, whereas C646 did not affect PKMζ expression, suggesting that PKMζ expression is possibly regulated by HDAC classes I and II, but not by p300. Memory research also found histone hyperacetylation enhanced PKMζ transcripts (Borodinova et al., 2019). However, more experiments need to be done to clarify the direct interaction of histone acetylation and PKMζ gene in ELS rats in the future.

A recent study found that the overexpression of neuron-specific HDAC2, but not that of HDAC1, reduced dendritic spine density, number of synapses, synaptic plasticity, and memory formation (Guan et al., 2009). On the contrary, HDAC2 deficiency resulted in an increase in the number of synapses and memory facilitation, similar to chronic treatment of HDAC in mice (Guan et al., 2009). The subtypes of HDAC plays different roles in synaptic plasticity and memory formation. Epigenetic modifications in the CeA serve as memories of adverse events that occurred during early life (Louwies and Greenwood-Van Meerveld, 2020). To clarify if high histone acetylation expression in ELS rats results from the decrease of HDAC expression, we examined HDAC1–3 expressions in the amygdala. It was found that HDAC1 and HDAC2 expressions decreased, whereas HDAC3 expression did not change significantly in the amygdala of ELS rats, indicating that the decreased HDAC1 and HDAC2 expression is responsible for the high level of histone acetylation in the amygdala of ELS rats. As a result, increased histone acetylation facilitated PKMζ expression in the CeA and caused visceral hypersensitivity in ELS rats. Recent literatures reported the existence of sexually dimorphic pain signaling in the spinal (Mapplebeck et al., 2018) and CeA (Louwies and Greenwood-Van Meerveld, 2020) of rats. Behavioral study showed estrous cycle and ELS are significant factors in visceral sensitivity (Moloney et al., 2016). In the future, it could be interesting to explore the sex difference in histone acetylation/PKMζ signaling in ELS rats induced by neonatal CRD.

In summary, we infer that neonatal CRD stimulation inhibited HDAC1 and HDAC2 expressions in the amygdala, which enhanced the level of histone acetylation in the amygdala of ELS rats. Modifying the levels of histone acetylation can affect visceral pain and anxiety-like behavior in rats. Moreover, PKMζ expression in the CeA was facilitated by increased histone acetylation, resulting in visceral hypersensitivity but not anxiety-like phenotype in ELS rats. Two potentially different molecular mechanisms might be involved in the downstream of increased histone acetylation modification in CeA that modulate visceral hypersensitivity and emotional comorbidity of ELS rats. Reprogramming inappropriate adaptations may result in sustaining extrinsic phenotypes, such as visceral hypersensitivity and anxiety-like behaviors in ELS rats. Since epigenetic programming is dynamic and revisable, reversing stress-induced abnormal epigenetic mechanisms may be crucial to relieve chronic symptoms in ELS rats.

## Conclusion

Early life stress are induced by nociceptive stimulation in neonatal rats, which might induce inappropriate reprogramming by decreasing HDAC1 and HDAC2 expressions in the amygdala. Increased acH3K9 is involved in the phenotypes of visceral hypersensitivity and anxiety-like behaviors in ELS rats. Furthermore, enhancing amygdala histone acetylation facilitates the expression of synaptic plasticity-related protein PKMζ, which regulates visceral hypersensitivity of ELS rats. This study could provide new ideas for the treatment of IBS.

## Data Availability Statement

The raw data supporting the conclusions of this article will be made available by the authors, without undue reservation.

## Ethics Statement

The animal study was reviewed and approved by the Committee for Care and Use of Laboratory Animals of Fujian Medical University.

## Author Contributions

LG: draft preparation, experiment design, *in vitro* experiments, and data analysis. XS and YT: behavioral experiments and data analysis. YY, LC, and GG: ELS model construct. YC: data analysis. CL and AC: experiment design, supervision, editing, and writing the manuscript. All authors contributed to the article and approved the submitted version.

## Conflict of Interest

The authors declare that the research was conducted in the absence of any commercial or financial relationships that could be construed as a potential conflict of interest.

## Publisher’s Note

All claims expressed in this article are solely those of the authors and do not necessarily represent those of their affiliated organizations, or those of the publisher, the editors and the reviewers. Any product that may be evaluated in this article, or claim that may be made by its manufacturer, is not guaranteed or endorsed by the publisher.
